# Using the SNAP-Tag technology to easily measure and demonstrate apoptotic changes in cancer and blood cells with different dyes

**DOI:** 10.1371/journal.pone.0243286

**Published:** 2020-12-03

**Authors:** Mira Woitok, Elena Grieger, Olusiji A. Akinrinmade, Susanne Bethke, Anh Tuan Pham, Christoph Stein, Rolf Fendel, Rainer Fischer, Stefan Barth, Judith Niesen

**Affiliations:** 1 Fraunhofer Institute for Molecular Biology and Applied Ecology (IME), Aachen, Germany; 2 Institute of Molecular Biotechnology (Biology VII), RWTH Aachen University, Aachen, Germany; 3 Institute of Infectious Disease and Molecular Medicine, Department of Integrative Biomedical Sciences, University of Cape Town, Observatory, Cape Town, South Africa; 4 Institute of Tropical Medicine, University of Tübingen, Tübingen, Germany; 5 Department of Pediatric Hematology and Oncology, University Medical Centre Hamburg-Eppendorf, Hamburg, Germany; 6 Mildred Scheel Cancer Career Center HaTriCS4, University Medical Center Hamburg-Eppendorf, Hamburg, Germany; 7 Research Institute Children’s Cancer Center, Hamburg, Germany; Columbia University, UNITED STATES

## Abstract

*In vitro* and *ex vivo* development of novel therapeutic agents requires reliable and accurate analyses of the cell conditions they were preclinical tested for, such as apoptosis. The detection of apoptotic cells by annexin V (AV) coupled to fluorophores has often shown limitations in the choice of the dye due to interference with other fluorescent-labeled cell markers. The SNAP-tag technology is an easy, rapid and versatile method for functionalization of proteins and was therefore used for labeling AV with various fluorophores. We generated the fusion protein AV-SNAP and analyzed its capacity for the specific display of apoptotic cells in various assays with therapeutic agents. AV-SNAP showed an efficient coupling reaction with five different fluorescent dyes. Two selected fluorophores were tested with suspension, adherent and peripheral blood cells, treated by heat-shock or apoptosis-inducing therapeutic agents. Flow cytometry analysis of apoptotic cells revealed a strong visualization using AV-SNAP coupled to these two fluorophores exemplary, which was comparable to a commercial AV-Assay-kit. The combination of the apoptosis-specific binding protein AV with the SNAP-tag provides a novel solid method to facilitate protein labeling using several, easy to change, fluorescent dyes at once. It avoids high costs and allows an ordinary exchange of dyes and easier use of other fluorescent-labeled cell markers, which is of high interest for the preclinical testing of therapeutic agents in e.g. cancer research.

## Introduction

Programmed cell death, or apoptosis, is a natural physiologic process during cell development of aging or homeostasis of cells. It may also facilitate the removal of unwanted, e.g. damaged, cells, which may result from e.g. different immune reactions [1]. In cancer, dysregulated cell death is also common and hence used as effective therapeutic line of attack [2–4]. Apoptosis is demonstrated by typical morphological changes such as cell shrinkage, packed organelles and an increased density of the cytoplasm. This results in a reduction in cell volume and the typical forming of apoptotic bodies, called budding. Additionally, chromatin condensation and nuclear fragmentation can be observed in cells undergoing apoptosis [1, 3, 5]. At the beginning of the nineties, it was found that annexin V (AV) bound calcium-dependently to phospholipid bilayers. In addition, it was discovered that phosphatidylserine (PS), a phospholipid located on the inner leaflet of the cell membrane in normal cells, is exposed on the surface of cells in the early apoptotic phase, for the specific recognition by lymphocytes, in this case macrophages [6, 7]. Since AV binds specifically, calcium-dependently and with high affinity to PS, it was employed for monitoring apoptotic cells, for example in flow cytometry assays [8, 9]. Also in the early nineties, the first flow cytometry assay using AV conjugated to FITC was evaluated, showing that fluorophore-conjugated AV can be used to detect apoptotic changes in cells with this method/assay [10]. In addition to apoptosis, cell death may alternatively occur by necrosis. In this case, the cells act passively since necrosis represents cell death that is triggered by external factors, diseases, infections, or toxins [1, 11]. The mechanisms and morphologic characteristics of cells undergoing necrosis are cell swelling, an expansion of the endoplasmic reticulum, the formation of cytoplasmic vacuoles as well as blebs, swelling of lysosomes and possibly disruption of the cell membrane [1]. To distinguish early apoptotic cells from late apoptotic and necrotic cells, propidium iodide (PI) is used in many flow cytometry assays. PI intercalates into DNA and can or may bind the nucleus of these late apoptotic/necrotic cells whose cell membrane is disintegrated [8, 12]. For these reasons, flow cytometry based AV/PI assays are commonly used to measure apoptotic and necrotic changes in target cells, such as cancer cells, which are exemplarily treated with novel therapeutic agents [8, 13–17]. Fluorescence activated cell sorting (FACS) based AV/PI-assays can also be combined with other methods such as staining approaches to detect cell death signaling pathways or differences in cell morphology [2]. However, this has drawbacks, such as that the fluorophore-conjugated AV must be exchanged if the fluorophore irradiates with other dyes used to demonstrate changes in cell morphology or cell pathways. Therefore, we used a novel technique by which the fluorophore conjugated to AV-SNAP can be easily exchanged, which makes it easy to use and include or combine other accessory staining methods. For this purpose, we employed the well-established SNAP-tag technology [18–23]. The SNAP-tag is a self-labeling protein-tag, which allows a rapid and simple labelling reaction with a variety of benzylguanine (BG)-modified dyes or fluorophores with various wavelengths [19–21, 24–26]. In previous studies, we could demonstrate the successful secretion and production of recombinant AV in a mammalian expression system [8]. By recombinantly fusing the AV-gene to the SNAP-tag and subsequent heterologous expression, high yields of recombinant and stable AV-SNAP fusion protein could be produced. This construct, coupled to a variety of different fluorophores, could then be utilized as flow cytometry based agent for e.g. AV/PI-assays. Using a protein for detection of PS has the advantage of less material waste and more stability for use in multiple comparable assays. We could demonstrate apoptotic changes using adherent and suspension cell lines, human peripheral blood mononuclear cells (PBMCs) as well as *ex vivo* differentiated macrophages. This was exemplarily demonstrated by using/combining two different dyes with our new established AV-SNAP fusion protein. This fusion protein is now available to use in apoptosis assays with different target cells, circumventing the above-mentioned drawbacks of standard and commercial available AV-staining procedures. Furthermore, it could be established also in other fluorescent-based cell assays e.g in cancer research.

## Materials and methods

### Cell lines and cell culture conditions

The human embryonic kidney cell line HEK 293T (CRL-11268), the human melanoma cell line A2058 (CRL-11147), the human mamma carcinoma cell line MDA-MB-468 (HTB-132), the human promyelocytic leukemia cell line HL-60 (CCL-240, kindly provided by the Department of Immunology, University Medical Centre Utrecht) and the human embryonal rhabdomyosarcoma cell line RD (CCL-136), were obtained from ATCC (American Type Culture Collection, Manassas, Virginia, USA). The T-cell leukemia cell line Jurkat (DSMZ no. ACC282) and the human epidermoid carcinoma cell line A431 (DSMZ no. ACC91) were obtained from the German Collection of Microorganism and Cell Culture (DSMZ, Braunschweig, Germany). All cell lines were cultivated in RPMI 1640 medium with GlutaMAX^™^, supplemented with 10% fetal calf serum (v/v) and 100 μg/ml penicillin and streptomycin (Gibco Invitrogen, CA, USA). Cell lines were cultivated under standard conditions at 37°C in a humidified atmosphere with 5% CO_2_. Zeocin (100 μg/ml) was added to the transfected HEK 293T cells for selection after transfection. All cell lines were authenticated by the analysis of short tandem repeats (STRs) and mycoplasma testing was done on a regular basis. PBMCs were separated from citrate-buffered peripheral blood by density gradient centrifugation using LSM Lymphocyte (GE Healthcare, Freiburg, Germany). The blood was donated by healthy human volunteers (University Clinic RWTH Aachen, Germany) and received from the blood bank oft he Transfusion Medicine Unit of the University University Clinic RWTH Aachen, Germany and with the approval of the Clinical Research Ethics Board of the University of Aachen. For the experiments, ‚Selective induction in M1 macrophages’ PBMCs were separated from citrate-buffered peripheral blood bags received from the Western Cape Blood Service, Cape Town, South Africa. The protocol was approved before commencement of study by the Human research ethics committee, faculty of health sciences, University of Cape Town, South Africa (approval number: 353/2017). The research only involved the use of healthy blood bags from the Western Cape Blood Service. Hence, the recruitment of study participants was not applicable. The need for informed consent was hereby waived by the human research ethics committee.

### Expression of AV-SNAP fusion protein

The AV-SNAP fusion protein was generated by introducing the previously described sequence of AV at the SfiI and NotI restriction sites of the pMS vector system (a derivate of the pSECTag2B vector from Invitrogen), which contains the SNAP-tag sequence [8, 21]. The fusion protein contains an N-terminal His_6_-tag and was transiently expressed in HEK 293T cells and purified by immobilized metal-ion affinity chromatography (IMAC) from cell culture supernatant using the His-tag, as previously described [20, 21]. SDS-PAGE and Coomassie Brilliant Blue staining verified the successful protein purification.

### Labelling of AV-SNAP

The purified fusion protein was incubated with 1.5-fold molar excess of the BG-modified fluorophores SNAP-Surface^®^AlexaFluor^®^488/505/532/647 (BG-488, BG-505, BG-532, BG-647); obtained from NEB (Ipswich, MA, USA) for 2 h at room temperature in the dark. The successful coupling of the fluorophores was verified by SDS-PAGE, followed by visualization of the fluorescence signals using a CRi Maestro system v2.2 (Cambridge Research Instrumentation, Inc. Woburn, MA, USA). The dye spectra were unmixed using the corresponding software 2.2. Excess fluorophores were removed using ZebaSpin^™^ desalting columns with a molecular weight cut-off (MWCO) of 40 kDa according to the manufacturer’s instruction (Thermo Fisher Scientific. Schwerte, Germany). To determine the coupling efficiency, the theoretical extinction coefficient of the protein and the extinction coefficients of the fluorescence dyes were used. The absorbance in solution of the conjugated protein at 280 nm and the excitation wavelength of the respective dye were measured using a photo spectrometer. Coupling efficacy was therefore defined photometrically (Biophotometer, Eppendorf, Hamburg, Germany) due to manufactures’ instructions (NEB, SNAP-Surface^®^ dyes), detailed explanation is presented in the S1 File and in the S1 Table in S1 File.

### Measurement of apoptotic effects

The induction of apoptosis in the cell lines by camptothecin, an antigen-specific antibody-drug conjugate (ADC), immunotoxins (IT) or targeted human cytolytic fusion proteins (hCFP) was measured by AV/PI staining as previously described [13–15, 27, 28]. The AV/PI staining protocol was optimized using two different fluorophore-coupled AV-SNAP fusion proteins instead of AV-eGFP supernatant or commercial available AV [14, 15, 29]. The fluorochromes AlexaFluor^®^ 488 and AlexaFluor^®^ 647 were selected since they are the most common used fluorochromes. They can be visualized in fluorescent channels which are mostly and standardized used in apoptosis assays in laboratory settings and are in most cases part of standard flow cytometry instruments. Briefly, cells were washed twice with AV binding buffer (10 mM HEPES/NaOH pH 7.4, 140 mM NaCl, 2.5 mM CaCl_2_) by centrifugation (500 × g, 5 min, room temperature) and stained with 0.5 μg AV-SNAP either coupled with BG-488 or BG-647 in 50 μl AV binding buffer for 20 min at room temperature in the dark. After a washing and centrifugation step, cells were suspended in AV binding buffer supplemented with 2 μg/ml PI and analyzed by flow cytometry using a FACSVerse instrument (Becton Dickinson, Heidelberg, Germany). For comparison cells were also stained with commercial available AnnexinV-FITC (e Bioscience, Frankfurt, Germany) using the same conditions as previously described.

### General induction of apoptosis by heat shock

Apoptosis induced by intense heat shock/stress, was analyzed after harvesting 2 × 10^6^ cells in 6-well plates and incubating them with either PBS (negative control) or camptothecin (positive control) for 4 h, at standard conditions or by heat treatment of the cells at 56°C for 30 min, following an incubation step for 4 h. Afterwards, the apoptotic effects were analyzed by AV-SNAP/PI staining, as described above. The apoptotic effects were evaluated by plotting the PerCP-Cy5-channel (FL-3/PE) against the FITC-channel (FL-1/488) for BG-488 or APC-channel (FL-4/APC) against PE-channel (FL-2/PE) for BG-647. All experiments were carried out in duplicate or triplicate. As before, cells were also stained with commercial available AnnexinV-FITC (eBioscience, Frankfurt, Germany) for comparison using the same conditions.

### Induction of apoptosis by antigen-specific fusion proteins

Briefly, at least 2.5 × 10^5^ EGFR- expressing target cells (MDA-MB-468, RD and A431) or CD33-expressing cells (HL-60) were seeded out in 24-well plates, incubated with either 10 nM EGFR-specific ADC [15], 15 nM EGFR-, CD33- or CD64-specific IT [30–32], PBS as negative control or 5 μM camptothecin as positive control for 72 h at standard conditions, before analysis of apoptotic effects by AV-SNAP/PI staining as described. EGFR^-^ A2058 cells were used as control cell line for the analysis of no unspecific apoptosis induction by the EGFR-specific ADC. For the induction of apoptosis by the different ITs EGFR^+^/CD64^-^ A431 and EGFR^-^/CD33^+-^ HL60 cells were used.

### Selective induction in M1 macrophages

Isolation, polarization and treatment of macrophages with a CD64-specific IT or a corresponding human cytolytic fusion protein (hCFP) was carried out as previously described [33, 34]. Briefly, PBMCs were isolated from buffy coats (Western Province Blood Transfusion Service, Cape Town, South Africa) and cultured for 3 hours in serum free media to select for monocytes by adherence. Monocytes were afterwards seeded at 1 x 10^5^ cells/well in 12 well plates and polarized for 72 h using 100 U/ml human IFN-γ (Sigma-Aldrich, St. Louis, MO, USA) and 1 μg/ml LPS (Sigma-Aldrich) for M1 and 20 ng/ml human IL-4 (Peprotech, Hamburg, Germany) for M2 macrophages. The polarization was boosted after 72 h with half of the initial stimulus for 24 h. Successful polarization of macrophages was confirmed by microscopic observation of cell morphology and fluorescent analysis of cell surface receptors. Thereafter, polarized macrophages were incubated with 200 nM hCFP and 100 nM IT. Negative control cells were treated with PBS. After 24 h, apoptosis assay was carried out as described above except that PI was replaced with 7-amino-actinomycin D (7-AAD) as a marker of viable cells. Before commencement of study, the protocol was approved by the Human ethics research committee of the University of Cape Town, South Africa.

## Results

### Expression, purification and labelling of AV-SNAP

**Fig 1:**
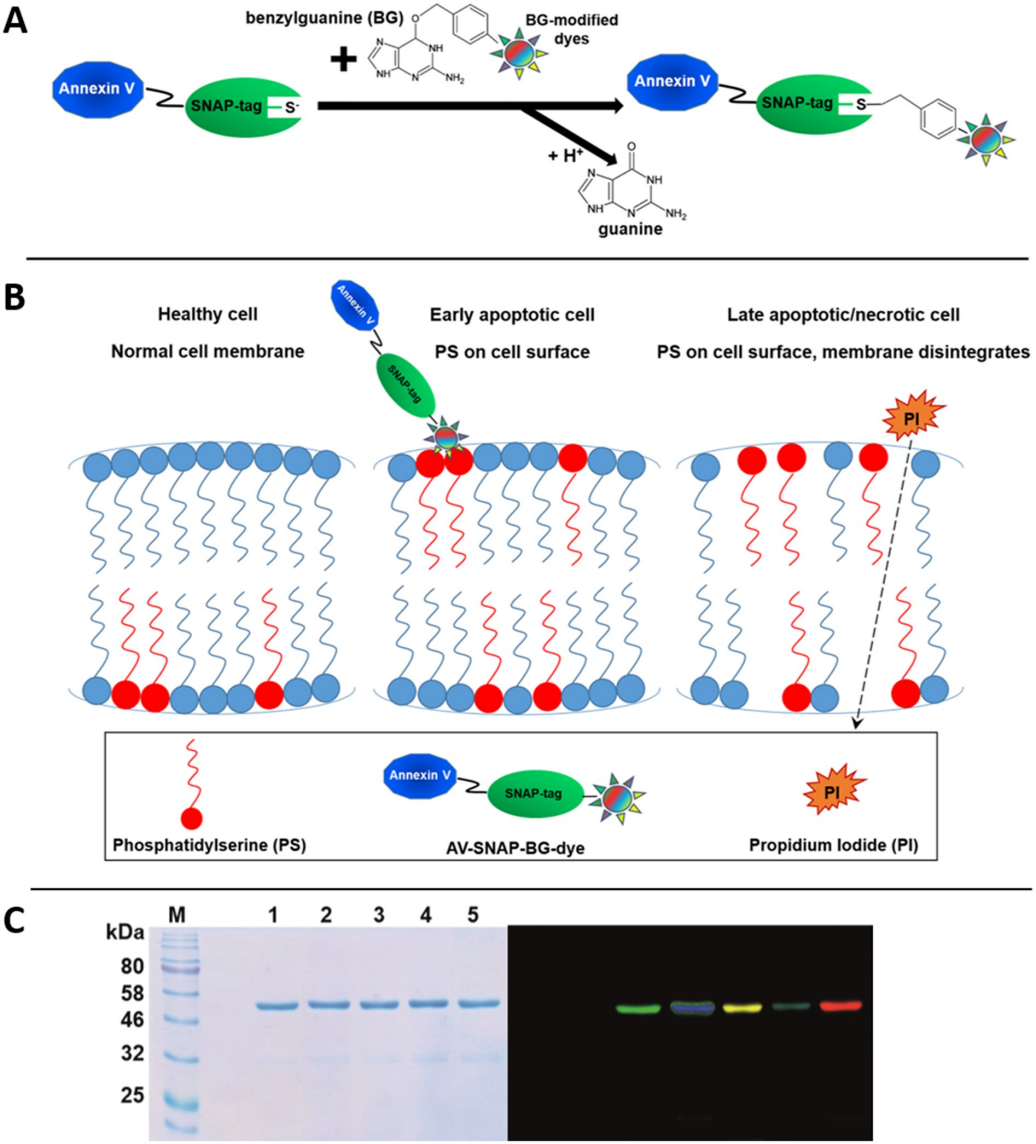
Schematic illustration of the functional characteristics of annexin V-SNAP (AV-SNAP) and SDS-PAGE of AV-SNAP coupled to various BG-modified dyes. (**A**) Simple and rapid labeling reaction of AV-SNAP to different BG-modified fluorescent dyes. The SNAP-tag technology demonstrates irreversible coupling of BG-modified dyes to AV-SNAP as a fusion protein. The labeling takes place in a 1:1 stoichiometry without inhibiting the fusion partner AV. During the labeling reaction, free guanine is released. (**B**) The principles of an AV apoptosis assay are demonstrated. On healthy cells with a normal cell membrane, phosphatidylserine (PS) is located on the inner surface. Phospholipids of the cell membrane are asymmetrically distributed between outer and inner cell membrane. During early stages of apoptosis, PS becomes exposed on the outer surface of the plasma cell membrane due to loss of plasma membrane asymmetry. PS exposed to the external cellular environment can be bound by AV which show high respective affinities and consequently the AV-SNAP-BG-dye coupled fusion protein can be used to detect apoptotic changes in target cells. In later stages of cell death, loss of membrane integrity occurs also, demonstrating late apoptotic as well as necrotic stages in cells. Typically, PI is used together with AV in the same assay, because PI is a vital dye and permeable cell membranes of damaged or dead cells are open for PI. **(C)** SDS-PAGE analysis of BG-fluorophore-labeled AV-SNAP. Coomassie Brilliant Blue staining (left) of the SDS PAGE and visualization of labeled fluorophores using the CRi Maestro imaging system (right) with the blue (500–720 nm) and yellow (630–850 nm) filter set. Dye spectra were unmixed using the associated software 2.2. (M) Color pre-stained protein standard; AV-SNAP labeled with (1) BG- 488, (2) BG-505, (3) BG-532, (4) BG-546 and (5) BG-647.

The SNAP-tag, derived from the human DNA-repair enzyme alkylguanine-DNA alkyltransferase is able to bind covalently and rapidly substrates containing O(6)-benzylguanine (BG). Hence, as a fusion partner, with e.g. AV, the SNAP-tag reaches irreversible coupling to BG-modifies substrates (Fig 1A). AV, an endogenous human protein, binds specifically to phosphatidylserine (PS) and can therefore be used, modified with fluorescent dyes, to detect apoptotic cells (Fig 1B). AV-SNAP fusion protein was expressed in HEK 293T cells and purified by IMAC, yielding 30 mg of purified fusion protein per liter of cell culture supernatant with a final purity of ~90%. Coomassie Brilliant Blue staining of the SDS-gel showed equal amounts of each coupled fusion protein loaded on the SDS-PAGE (Fig 1C). To verify SNAP tag activity, several BG-modified fluorophores were coupled to the purified fusion protein. The successful coupling of BG-488, BG-505, BG-532, BG-546 and BG-647 was analyzed by SDS-PAGE followed by visualization of the different fluorophores using a CRi Maestro Imaging device (Fig 1C). Coupling efficacies up to ~80% were reached by incubation of the AV-SNAP protein with a 1.5-fold molar excess of dye to protein.

### Induction of apoptosis by antigen-specific fusion proteins

**Fig 2:**
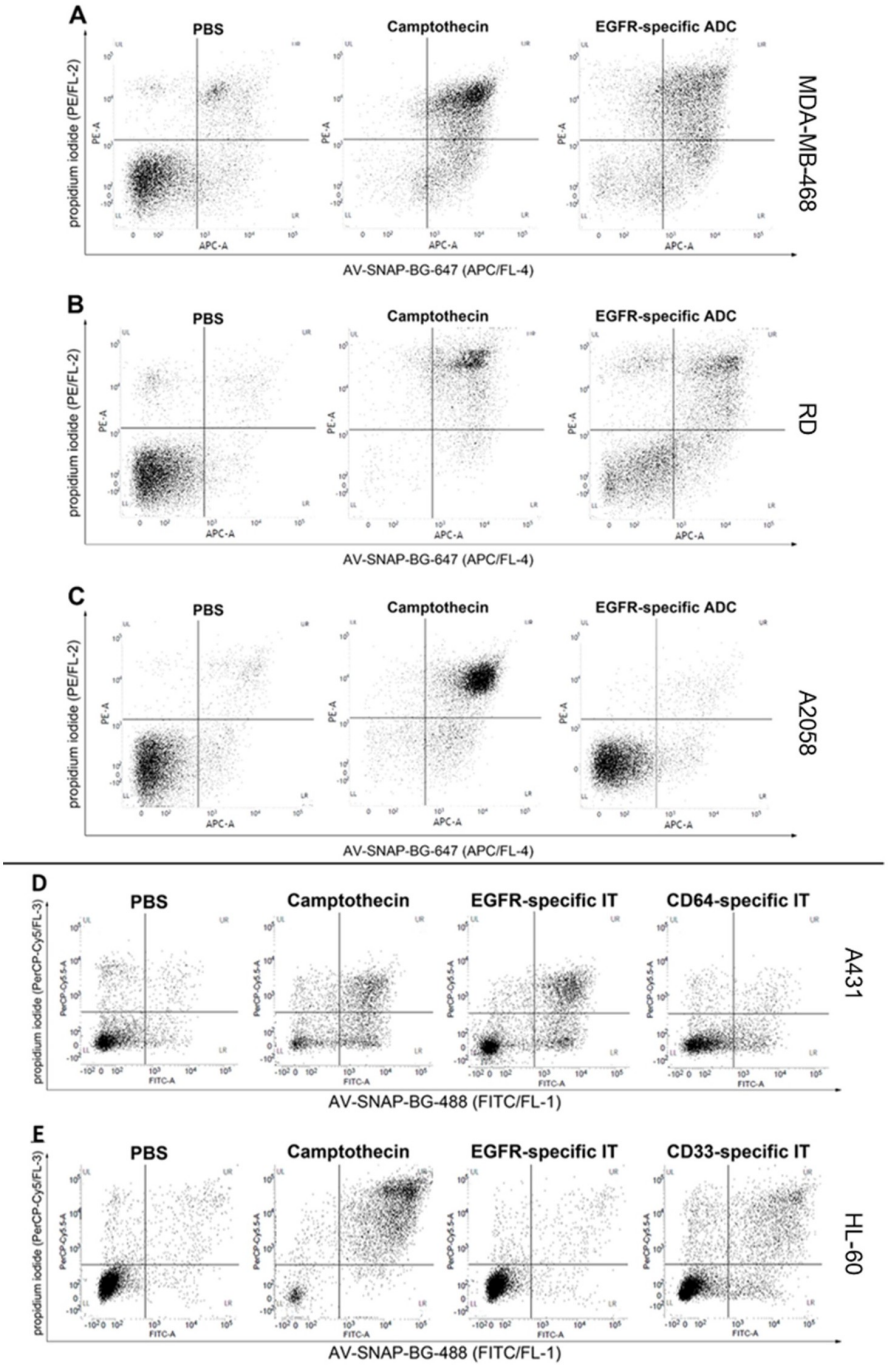
Analysis of apoptosis induction by an EGFR-specific ADC and different ITs using AV-SNAP-BG-647/PI or AV-SNAP-BG-488/PI staining. AV-SNAP-BG-647 for visualization of apoptotic cells induced by an EGFR-specific ADC. Representative dot plots of EGFR^+^ MDA-MB-468 (**A**), EGFR^+^ RD (**B**) or EGFR^-^ A2058 (**C**) cells either treated with PBS, 5 μM camptothecin or 10 nM of an EGFR-specific ADC for 72 h at 37°C and 5% CO_2_. Cells were stained with AV-SNAP-BG-647/PI and analyzed by flow cytometry. Experiments were carried out in triplicate (lower left quadrant viable cells, lower right quadrant: early apoptotic cells, upper right quadrant: late apoptotic/necrotic cells). **(D**-**E**) Demonstration of apoptotic effects induced by antigen-specific ITs using AV-SNAP-BG-488 for visualization. Dot plots of AV-SNAP-BG-488/PI stained A431 (adherent) (**D**) and HL-60 cells (in suspension) (**E**) treated either with PBS, 5 μM camptothecin, an EGFR-specific IT or a CD64-specific IT, 15 nM each (**D**, negative control) or a CD33-specific IT (**E**, positive control) for 72 h. Analysis was carried out by flow cytometry in the appropriate channels (FL-1 and FL-4).

Specific induction of apoptosis by an EGFR-specific ADC in adherent EGFR-expressing target cancer cells was analyzed using AV-SNAP-BG-647/PI staining (Fig 2A–2C). AV-SNAP-BG-488 was used to stain apoptotic cells of adherent EGFR^+^/CD64^-^ A431 cells and suspension EGFR^-^/CD33^+^ HL-60 cells treated with an EGFR-specific or a CD33/CD64-specific IT (Fig 2D and 2E). All cell lines showed increased proportions of apoptotic/necrotic cells after treatment with the ADCs or ITs (Fig 2A–2D; lower left corner: viable cells, lower right corner: early apoptotic cells, upper right corner: late apoptotic/necrotic cells) after treatment with camptothecin compared to PBS treated control cells. Treatment of the EGFR^+^ target cell lines MDA-MB-468 and RD with the EGFR-specific ADC also lead to a distinct increase in early as well as late apoptotic/necrotic cells (Fig 2A and 2B), whereby EGFR^-^ A2058 cells were not impaired by the ADC as compared to PBS-treated cells (Fig 2C). The EGFR-specific IT and the CD33-specific IT also increased the apoptotic/necrotic cell proportion on A431 and HL-60 cells, respectively (Fig 2D and 2E). As expected A431 cells were not affected by the CD64-specific IT (Fig 2D), and the EGFR-specific IT showed no impact on HL-60 cells (Fig 2E).

### Induction of apoptosis by heat shock

**Fig 3:**
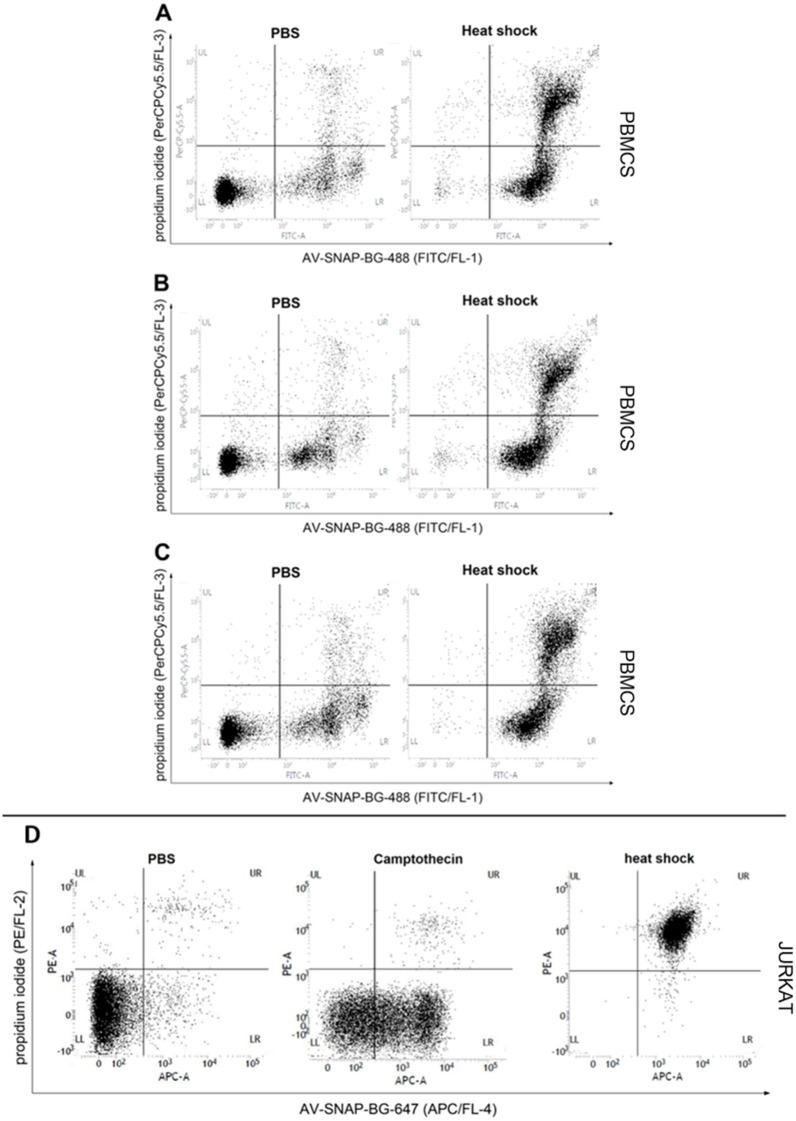
Detection of heat shock induced apoptosis in human PBMCs using AV-SNAP-BG-488 and PI staining and in suspension cells using AV-SNAP-BG-647 and PI staining. AV-SNAP-BG-488 was used to demonstrate apoptotic changes in human peripheral blood cells (PBMCs) induced by heat shock. Apoptotic effects after heat shock incubation at 56°C for 30 min was compared to PBS on PBMCs of three different donors (**A, B, C**) and analyzed after 4 h incubation at 37°C in a flow cytometry assay by AV-SNAP-BG-488/PI staining. **(D)** AV-SNAP-BG-647 was used to visualize apoptosis in suspension cells induced by heat shock. Jurkat cells were treated either with PBS, 5 μM camptothecin or a heat shock. Apoptotic cells are visualized by AV-SNAP-BG-647 and PI staining and analyzed by flow cytometry.

Visualization of apoptotic/necrotic cells in human PBMCs (Fig 3A–3C) or in the suspension cell line Jurkat (Fig 3D) was carried out using a “heat shock assay”. Heat-shock treated PMBCs showed a high proportion of early apoptotic and late apoptotic/necrotic cells compared to PBS-treated cells after staining with AV-SNAP-BG-488 (Fig 3A–3C). Comparing all three donors (Fig 3A–3C), no differences could be observed. Because of the harsh conditions for PBMCs in this assay, camptothecin treatment was skipped.

Jurkat cells treated with camptothecin showed a distinct increase of early apoptotic or late apoptotic/necrotic cells after AV-SNAP-BG-647/PI staining compared to PBS-treated cells (Fig 3D). A high amount of late apoptotic cells could be observed after heat shock induction (Fig 3D). After heat shock treatment, almost the complete cell population could be detected in the upper right quadrant of the dot plot, indicating the high proportion of late apoptotic/necrotic cells (Fig 3D, 3^rd^ dot blot). Treatment with camptothecin for 4 h resulted in an increase in the early apoptotic cell population (Fig 3D, lower right quadrant).

### Selective induction of apoptosis in M1 macrophages

**Fig 4:**
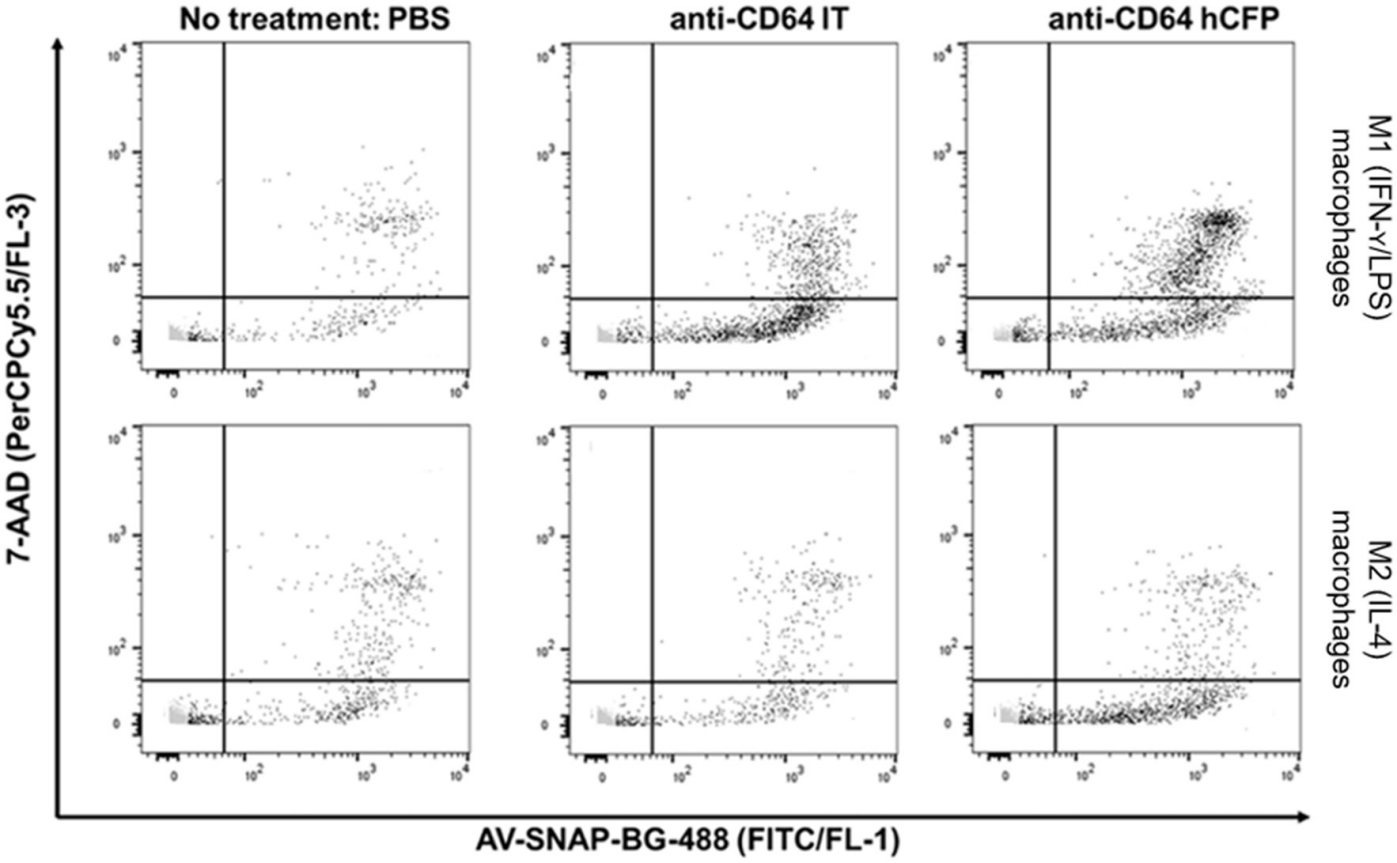
Induction of apoptosis in *ex vivo* differentiated human M1(IFN-γ/LPS) macrophages using AV-SNAP-BG-488 and 7-AAD staining. Visualization of the selective induction of apoptosis by a CD64-specific IT or hCFP using AV-SNAP-BG-488/7-AAD staining on *ex vivo* differentiated human M1 (IFN-γ/LPS) macrophages. Analysis was carried out using flow cytometry by plotting fluorescence channel FL-1 (FITC/AV-SNAP-BG-488) against FL-3 (PerCPCy5.5/7-AAD).

The successful polarization of human macrophages into phenotypic sub-types was confirmed by microscopic observations and flow cytometric analysis of cell surface receptors. The morphological features of M1 (IFN-γ & LPS) and M2 (IL-4) human macrophages as observed by fluorescent microscopy (S2A Fig). The M1 (IFN-γ & LPS) polarized macrophages presented more of a spindle shape morphology with some cells appearing round-elongated. On the other hand, the M2 (IL-4) macrophages exhibited a more spread / round morphology when compared to the M1 (IFN-γ & LPS) differentiated macrophages. Next, the expression of membrane polarization markers was assayed by flow cytometry. CD14 was highly expressed on M1 (IFN-γ & LPS) but not on M2 (IL-4) macrophages (S2B Fig) while the macrophage mannose receptor (CD206) commonly associated with M2 (IL-4) polarization was highly expressed in M2-type macrophages when compared with the M1 (IFN-γ & LPS) macrophages. Upon confirmation of successful polarization of macrophages, the macrophages were treated with apoptosis inducing antibody based fusion proteins. The use of recombinant CD64-specific IT to selectively kill pro-inflammatory M1- (IFN-γ/LPS) but not anti-inflammatory M2 (IL-4) macrophages, has been previously demonstrated in different *in vitro*, *in vivo* and *ex vivo* models [34]. In this study, AV-SNAP-BG-488 was used to monitor selective induction of apoptosis of *ex vivo* differentiated M1 (IFN-γ/LPS) macrophages by a CD64-specific IT and a corresponding hCFP (Fig 4). Late apoptotic cells were visualized by simultaneous 7-AAD staining instead of PI staining. Data were acquired by flow cytometry, plotting fluorescence channel FL-1 against FL-3. As expected, only M1 macrophages showed a distinct shift to the upper right quadrant of the dot plot, representing cells in the late apoptotic stage (Fig 4).

### Comparison of AV-SNAP with commercial available labeled AV

**Fig 5:**
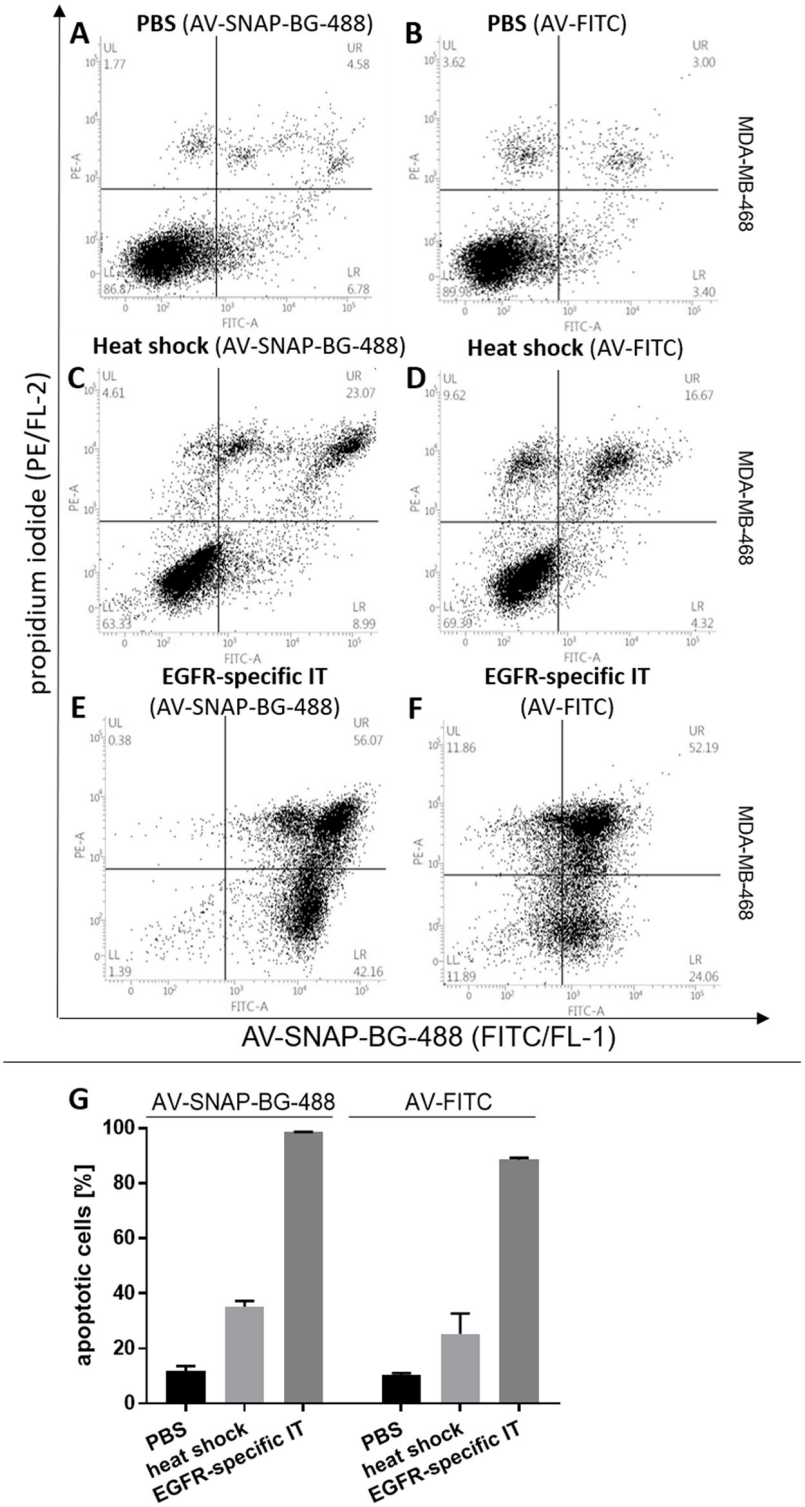
Comparison of apoptotic effects in MDA-MB-468 cells using AV-SNAP-BG-488 and AV-FITC. AV-SNAP-BG-488 **(A, C, E)** was used for visualization of apoptotic cells induced by an EGFR-specific IT or heat shock compared to AV-FITC (eBioscience) **(B, D, F)**. Representative dot plots are shown of EGFR^+^ MDA-MB-468 cells either treated with PBS, 15 nM of an EGFR-specific IT or heat shock **(A-F)**. **(G)** Grouped bar plot demonstrates the comparable percent of early and late apoptotic cells measured with AV-SNAP-BG-488 or AV-FITC by flow cytometry.

By way of comparison, exemplarily the AV-SNAP-BG-488 was compared to a commercial available AV-FITC-kit (eBioscience, Frankfurt, Germany). MDA-MB-468 cells were treated with PBS as control (Fig 5A and 5B), with heat-shock as previously described (Fig 5C and 5D) and with an EGFR-specific IT (Fig 5E and 5F) to measure and compare the apoptotic changes in the cells using both AV-agents. AV-SNAP-BG-488 and the commercial AV-FITC demonstrated comparable results. To validate the comparable apoptotic effects, three independent experiments are demonstrated in a grouped bar plot in Fig 5G.

## Discussion

To this day, an AV-assay provides an efficient method for the evaluation by an accurate detection of apoptotic cells in their different stages of apoptosis [10]. It is a well-established cellular *in vitro* assay, that is often used in various areas of research e.g., in the development of novel anti-cancer therapeutics [9, 13–15, 17, 35, 36]. Often, commercial available chemical-labeled fluorescent AV is also used in the form of detection kits [17, 35]. Diverse distributors offer AV with different coupled fluorophores to provide the best solutions for the individual assay. In addition, genetically fluorescent AV-fusions like AV-eGFP, that can be easily expressed in eukaryotic cell systems, were used in AV-assays not only as purified protein but also as expressing cell culture supernatant [8]. Nevertheless, both variants show limitation for the operator, such as availability of only one fluorophore in the laboratory, or the costly purchase of different AV-fluorophore variants for the different applications. Furthermore, chemical labeling of AV with fluorophores results in a heterogeneous mixture of relatively photolabile molecules [8]. To allow homogenous fluorophore coupling, we generated an AV-SNAP fusion protein that allows fast, efficient and covalent coupling of different BG-modified fluorophores with a defined stoichiometry, using the SNAP-tag technology. The SNAP-tag is a self-labeling protein tag that reacts enzymatically with BG [19, 20, 37]. It is widely used for fluorescent labeling of fusion proteins and bio-functionalization of surfaces [38] or nanoparticles [39]. Furthermore, scFv-SNAP-based fusion proteins have been used to establish *in vivo* imaging probes [40] or novel ADCs by coupling of BG-modified effector molecules such as photosensitizers or auristatin F [15, 41]. In several studies, it could be demonstrated that the defined coupling of BG-modified molecules did not alter the binding capacity of the fusion protein [19–21].

We could express the fusion protein AV-SNAP in HEK293T cells, and purified it from cell culture supernatant by IMAC with a yield of ~30 mg/L cell culture supernatant and a purity of ~90%. The functionality of the SNAP-tag in AV-SNAP was demonstrated by coupling it to five different fluorophores. An incubation time of 2 hours and 1.5 molar excess of BG-modified dye to protein was sufficient to demonstrate an equal coupling efficiency of the samples. Therefore, when AV-SNAP is expressed and purified, coupling can be conducted in a short period and the protein is ready to use after a brief centrifugation step to get rid of the unbound dye. The availability of a broad range of dyes combined with the rapid and easy ready-to-use technique for functionalizing proteins, makes the SNAP-tag technology a useful tool for this purpose [18, 20, 40]. The flexibility in the choice of fluorophores is not trivial since some fluorophores might irradiate with other dyes (e.g. nuclear staining dyes) in the emission spectra, resulting in undesired compensation settings or background fluorescence [42].

We exemplarily chose/selected two frequently used fluorophores (BG-488 and BG-647) coupled them to AV-SNAP, and further characterized their potential in three different apoptosis induction methods, using cells with different characteristics such as suspension cells, adherent cells, and human PBMCs. In all cell types, apoptosis induction by different compounds could be successfully measured using AV-SNAP coupled to a fluorophore.

Commercially available detection kits are often only tested with suspension cells and do not yield sufficient results when using them with adherent cells or PBMCs, a problem that we faced several times in our laboratory. To that effect, we chose suspension and adherent cancer cell lines, and additionally isolated human peripheral blood cells to compare staining effectiveness of the novel AV-SNAP based fusion proteins. The selected cell lines, including A2058, A431, MDA-MD-468, RD, Jurkat, and HL-60, are well-used cell lines for research and drug development, commonly available and characterized by their overexpression of special cell markers. Furthermore, we utilized different apoptosis induction methods by using well characterized ITs, recombinant ADCs, known apoptosis inducing agents or heat shock treatment to demonstrate the suitability of AV-SNAP for diverse assay conditions. Under all conditions, positive and expected apoptosis induction could be observed, demonstrating that our newly established AV-SNAP fusion partner is an excellent module to measure apoptosis in different types of cells with a wide range of fluorophores, which could be easily and rapidly exchanged for the particular purpose [18, 20]. As demonstrated, AV-SNAP-BG-488 shows comparable results to a commercial available AV-FITC-kit and can be used equivalent.

The efficacy of apoptosis detection using the novel AV-SNAP-BG-647 fusion proteins was assessed on adherent EGFR-overexpressing cancer cell lines treated with an EGFR-specific ADC, as well as on suspension cells (Jurkat cells) after heat shock treatment. As expected in both experiments, a major shift of the cell population in the lower right and/or upper right quadrant could be detected. Treatment with camptothecin was carried out as positive control and apoptosis induction was more pronounced in assays with longer incubation times. Camptothecin is a well-characterized apoptosis inducing agent and often used as positive control [43, 44]. Furthermore, heat shock treatment is a widely-used control method since eukaryotic cells form misfolded or aggregated proteins in the presence of thermal insults, thus inducing apoptosis [45, 46].

In addition, the suitability of AV-SNAP-BG-488 could be verified in three different settings. We could demonstrate effective visualization of apoptosis induction when using well characterized ITs in adherent and suspension cells, by heat shock treatment in PBMCs, as well as by using characterized ITs and hCFPs to selectively kill *ex vivo* differentiated human M1 (IFN-Y/LPS) versus M2 (IL-4) macrophages. When comparing PBMCs from three different donors, no significant deviations are detectable, thus reflecting the good reproducibility of the assay conditions.

Both coupled AV-SNAP fusion proteins could be used in combination with e.g. PI or 7-AAD to clearly distinguish apoptotic and healthy cells, regardless of whether cells were adherent or in suspension or in which way apoptosis was induced. For both coupled AV-based fusion proteins, an amount of 1 μg of coupled AV-SNAP was sufficient to ensure an efficient result and reliable reproducibility. The high expression yield and the fast and efficient labeling of AV-SNAP makes the fusion protein an attractive reagent, since often high amounts of AV is needed if experiments are carried out in replicates or different fluorophores are required.

Moreover, when exemplary using transfected cell lines expressing fluorescent reporter genes such as eGFP or mCherry, the choice of the right fluorophore coupled to AV is much more limited [47]. Additionally, a more specific and broader analysis of apoptotic cells regarding their cell cycle or division rate might be carried out simultaneously to the AV-assay. When using AV-SNAP, different fluorophores can be coupled easily and fast. Thus, varieties of BG-modified fluorophores are available or the desired fluorophore can easily be modified by using available BG-modified linker structures [19, 21].

In conclusion, we could demonstrate that the SNAP-Tag is a useful tool for *in vitro* and *ex vivo* analysis of apoptosis by using an AV-SNAP fusion protein due to the flexibility of fluorophore coupling. Furthermore, reliable and reproducible results could be obtained in varying assay conditions with different cell characteristics.

## Supporting information

S1 Fig (PDF)

S2 Fig **A:** Confocal fluorescence microscopy images showing cytoskeletal actin (Alexa Fluor^™^ 488 Phalloidin, green (Invitrogen)) and DAPI nuclear stain (blue) staining of M1 (IFN-γ & LPS), and M2 (IL-4) macrophages (a, and b). Images were taken with the LSM 880 Airy scan confocal microscope (Zeiss). **B:** Flow cytometric characterization of CD14 and CD206 surface receptors on M1 (IFN-γ & LPS) and M2 (IL-4), monocyte derived macrophages. Cells were stained for fluorescence activated cell sorting (FACS) analysis using Alexa 647 conjugated human anti-CD14 and anti-CD206 antibodies.
(TIF)

S1 File (DOCX)
