# Supplementary material for: Using the SNAP-Tag technology to easily measure and demonstrate apoptotic changes in cancer and blood cells with different dyes
Source: PLoS One. 2020 Dec 3;15(12):e0243286. doi: 10.1371/journal.pone.0243286 (PMC7714129; doi:10.1371/journal.pone.0243286)
Supplement: S1 Fig — (PDF) [file pone.0243286.s001.pdf]

A_1

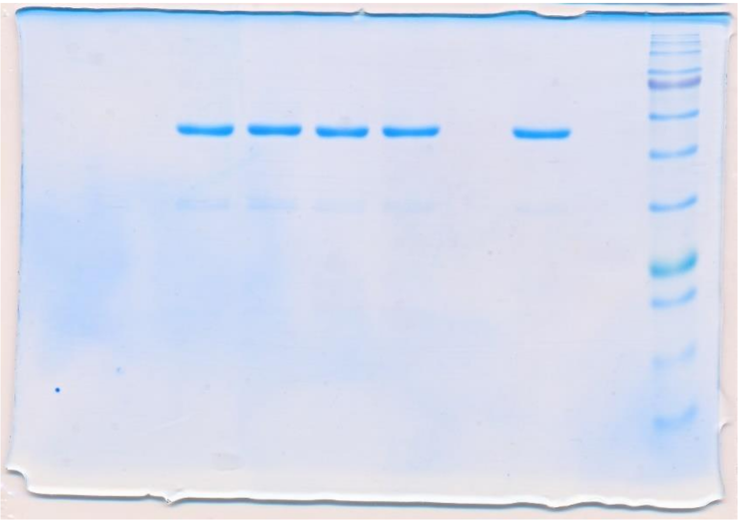

B

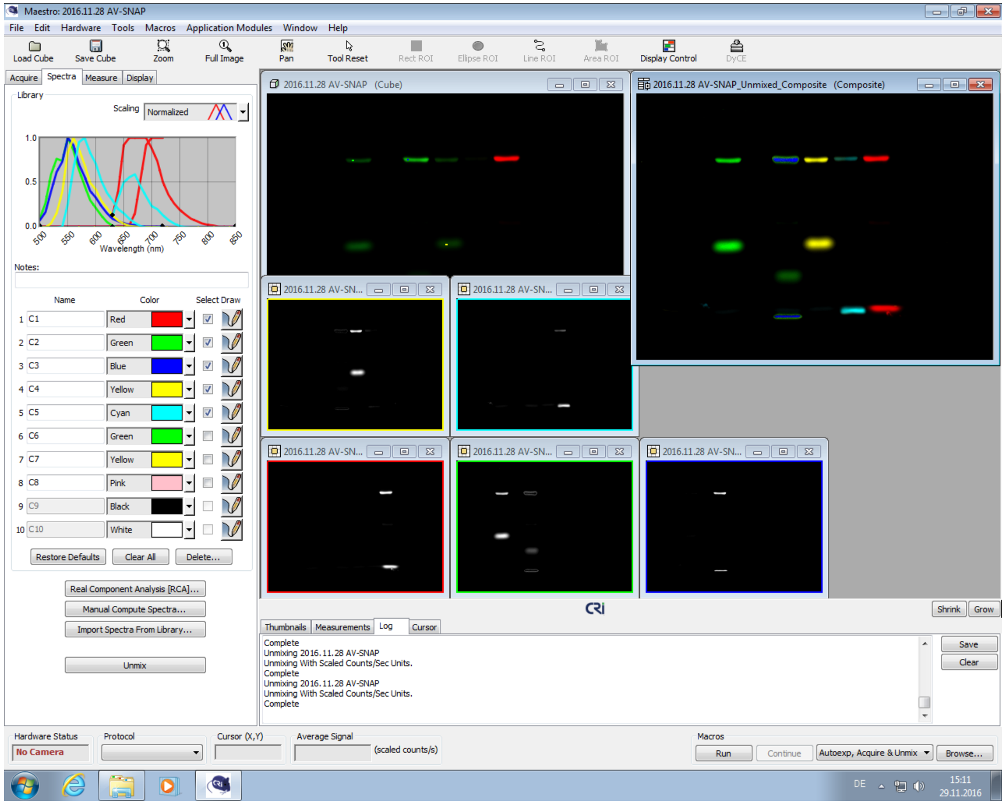

- Figure panel Fig.1 A was generated from that original image
- Molecular weight marker is shown, also in the Fig.1 in the manuscript itself
- Fig.1 A second part was generated from Fig. S1 B
