# Supplementary figures and images for: Using the SNAP-Tag technology to easily measure and demonstrate apoptotic changes in cancer and blood cells with different dyes
Source: PLoS One. 2020 Dec 3;15(12):e0243286. doi: 10.1371/journal.pone.0243286 (PMC7714129; doi:10.1371/journal.pone.0243286)

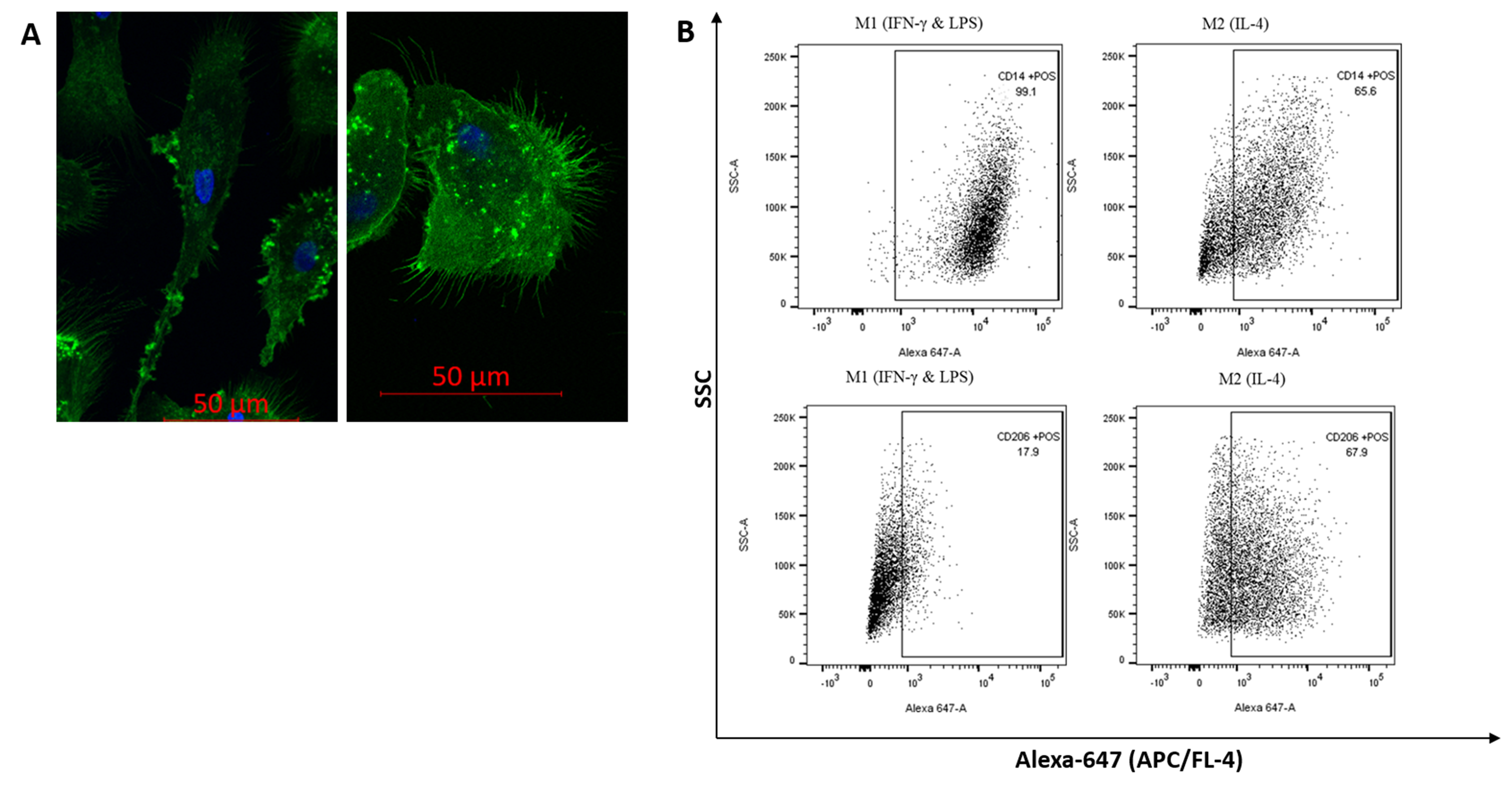

Supplement: S2 Fig — A: Confocal fluorescence microscopy images showing cytoskeletal actin (Alexa Fluor™ 488 Phalloidin, green (Invitrogen)) and DAPI nuclear stain (blue) staining of M1 (IFN-γ & LPS), and M2 (IL-4) macrophages (a, and b). Images were taken with the LSM 880 Airy scan confocal microscope (Zeiss). B: Flow cytometric characterization of CD14 and CD206 surface receptors on M1 (IFN-γ & LPS) and M2 (IL-4), monocyte derived macrophages. Cells were stained for fluorescence activated cell sorting (FACS) analysis using Alexa 647 conjugated human anti-CD14 and anti-CD206 antibodies. (TIF) [file pone.0243286.s002.tif]
