# Supplementary material for: Using the SNAP-Tag technology to easily measure and demonstrate apoptotic changes in cancer and blood cells with different dyes
Source: PLoS One. 2020 Dec 3;15(12):e0243286. doi: 10.1371/journal.pone.0243286 (PMC7714129; doi:10.1371/journal.pone.0243286)
Supplement: S1 File — (DOCX) [file pone.0243286.s003.docx]

**Supplementary material:**

***Determination of the degree of labelling of AV-SNAP-tag based constructs***

1. Take a sample of the labeled SNAP-tag fusion protein in a suitable buffer at pH 7.0 to 8.0 and measure the absorbance in a micro-cuvette with 1 cm path-length at 506 nm (*AlexaFluor^®^ 488*) or 650 nm (*AlexaFluor^®^ 647*) , and at 280 nm (protein reference)

- dilute sample to get an absorbance in the range from 0.1 to 1.5

2. Calculate the molar concentration of the label:

Label conc. = A650 or A506 × dilution factor/ 90000 or 239000

- 90000 M-1 cm-1 is the approximate molar extinction coefficient of *AlexaFluor^®^ 488* dye at 506 nm and 239000 M-1 cm-1 is the approximate molar extinction coefficient of *AlexaFluor^®^ 647* dye at 650 nm

3. Determine the molar concentration of protein in the sample:

- measure absorbance at 280 nm

- molar extinction coefficient of the SNAP-tag sequence is ≈ 21300 M-1 cm-1 and the molar extinction coefficient of AV-SNAP is 44350 M-1 cm-1

- calculate the protein concentration as follows:

Protein conc. = (A280 – (A650 x 0,549)) x dilution factor / combined extinction factor

- 0,549 is the absorbance of *AlexaFluor^®^ 647* at 280 nm as a proportion of its absorbance at 650 nm

Or

Protein conc. = (A280 – (A506 x 0,269)) x dilution factor / combined extinction factor

- 0,269 is the absorbance of *AlexaFluor^®^ 488* at 280 nm as a proportion of its absorbance at 506 nm

4. Calculate the degree of labeling:

Degree of labeling = Label conc./ Protein conc.

**Supp. Table 1:**

| ***AlexaFluor^®^ 488*** | Absorbance  (280nm) | Dilution factor | Absorbance Dye (506nm) | Dye absorbance  280nm (F) | Extinction coefficient dye | Extinction coefficient AV-SNAP |
| --- | --- | --- | --- | --- | --- | --- |
|  | 0,224 | 2 | 0,269 | 0,198 | 90000 | 44350 |
|  | | | | | | |
| Results: |  | | | | | |
| Label conc. | 5,97778E-06 |  |  |  |  |  |
| Protein conc. | 7,69957E-06 |  |  |  |  |  |
| **% labeling** | **~ 80%** |  |  |  |  |  |

| ***AlexaFluor^®^ 647*** | Absorbance  (280nm) | Dilution factor | Absorbance Dye (650nm) | Dye absorbance  280nm (F) | Extinction coefficient dye | Extinction coefficient AV-SNAP |
| --- | --- | --- | --- | --- | --- | --- |
|  | 0,195 | 3 | 0,549 | 0,13 | 239000 | 44350 |
|  | | | | | | |
| Results: |  | | | | | |
| Label conc. | 6,89121E-06 |  |  |  |  |  |
| Protein conc. | 8,3628E-06 |  |  |  |  |  |
| **% labeling** | **~ 82%** |  |  |  |  |  |
